# Progress in Bacteriology

**Published:** 1903-05-02

**Authors:** 


### Progress in Bacteriology.

### DIPHTHERIA.

Bellfanti and Vedova were the first observers to draw attention to the presence of diphtheria-like organisms in atrophic rhinitis. Symes [1] has examined 23 typical cases, and in 20 of these a bacillus was found which in morphological and cultural characteristics clearly resembles the diphtheria bacillus in its characteristic long-beaded form. The bacillus mucosus was present in 16 of the cases. The diphtheria-like bacillus was not present in healthy noses or in other diseased conditions. Whilst closely resembling the Klebs-Löffler bacillus, and giving rise to similar fatal results when injected into guinea-pigs, the micro-organism found in atrophic rhinitis has certain constant differences, of which the following are the chief:—The bacillus of atrophic rhinitis is thinner, more curved, and more highly metachromatic, and does not grow so readily on Kanthack's medium. The writer goes on to adduce other evidence why atrophic rhinitis should be regarded as a chronic form of diphtheria, and in view of the importance of this matter in connection with the spread of infection urges that further investigations should be made.

A recent paper by Lupnik [2] mentions the fact that typical diphtheria bacilli may be found in ozæna, and also in other conditions, such as xerosis, membranous rhinitis, noma, pemphigus vegetans, scarlet fever, tonsillitis, pneumonia, bronchitis and empyema This writer considers, indeed, that the Löffler bacillus has no specific etiological relationship to diphtheria, and he expresses the doubt as to whether diphtheria antitoxin is any more efficacious in an angina caused by the Löffler bacillus than in that caused by any other organism. In many cases of diphtheria no bacilli can be detected.

He divides diphtheria bacilli into two groups—the Hoffmann-Wellenhoff group (the pseudo-forms) and the Löffler group. The first are short plasmolysed bacilli, growing readily on agar, and arranged in stained films in parallel lines. They are to be found in air, urine, stools, and in the skin of men and animals; also they occur in such diseases as scarlet fever, conjunctivitis and actinomycosis. They are probably in no way related to the causation of diphtheria, and are not virulent to guinea-pigs. There are probably very many distinct varieties of Löffler bacilli, and they differ very widely in their cultural characteristics. Most of these long plasmolysed bacilli are, however, pathogenic to guinea-pigs, but all may undergo very rapid changes of character in a comparatively short space of time.

Wassermann [3] has attempted to produce a diphtheria serum which in addition to possessing antitoxic properties shall also have anti-bacterial action; that is to say, in addition to chemically neutralising the toxin in the body, the serum would precipitate and agglutinate the invading micro-organisms and finally dissolve them in virtue of its lysogenic properties. If such a serum could be obtained it was obvious that the destruction of the bacilli would materially shorten the period of necessary isolation. The serum was obtained as follows:—Diphtheria bacilli were dried for 24 hours at 60°C. and pulverised in an agate mortar. To each gramme of this powder there was added 20ccs. of ethylendiamin solution, and after some hours' shaking the solution was filtered and centrifugalised. The clear fluid so obtained contains an extract of the bodies of the bacilli; it also contains a certain amount of diphtheria toxin, but this is neutralised by the addition of the required amount of antitoxin. Rabbits injected with this neutralised serum developed antibacterial properties in their blood. Such a serum should theoretically prove of value in the treatment of the disease in man, and will in the laboratory be a useful means of distinguishing between true and pseudo-diphtheria bacilli; for on the latter its bactericidal power would be ineffective. Piorkowski's differential stain for the diphtheria bacillus was as follows:—Stain with Löffler's blue for 2 minutes; decolorise for 1 second with 3 per cent. hydrochloric acid, wash, counterstain for 10 seconds with a half per cent. aqueous solution of eosin. The bodies of the bacilli are stained a pale red colour, the polar bodies a dark blue-black. For this method of staining 24-hour cultures on Löffler's blood serum are most suitable. Schauffler [4] proposes a modification of this process, using one compound dye which is applied in the cold for one minute. His method is applicable to smears made direct from the fresh membrane and to agar cultures of from 24 hours' to 21 days' incubation. The composition of the stain is as follows:—Filtered solution of Löffler's methylene blue, 10cc.; filtered solution of pyronin (Grübler), 1·5ccs. (pyronin ·5 gram. aq. dist., 10cc.); 3 per cent. Hcl. alcohol 5cc. (alcohol absolute 97cc. Hcl. 25 per cent. 3cc.). The bodies of the bacilli are stained blue while the poles are a bright ruby red. Pseudo-diphtheria bacilli take on only a blue colour, and other bacilli such as those of cholera and plague show granular bodies, but these are not metachromatic.

Of bacteria closely allied to the diphtheria bacillus several have been recently described. One such variety associated with a case of puerperal fever was recorded by Foulerton and Bonney.[5] In most morphological characteristics it corresponded with the true Löffler's bacillus, but it was not pathogenic to guinea-pigs, did not form acid in glucose peptone broth, and was generally larger and coarser. It may be allied to the diphtheroid bacillus described in association with noma vulvæ, and other purulent conditions of the genital organs. Klein [6] has observed another diphtheroid bacillus in association with consolidation of the lungs in rats. Morphologically it resembles the Klebs-Löffler bacillus staining with Neisser's solution and forming acid from glucose. Inoculated into rats it produced a chronic abscess, but was never fatal. Diphtheria antitoxin did not neutralise the pathogenic action of a small dose of culture.

[1] Brit. Med. Jour., Feb. 28, 1903. [2] Prager Med. Woch. vol. xxvii., 1902. [3] Deut. Med. Woch., Oct. 31, 1902. [4] Med. Rec., Dec. 6, 1902. [5] Brit. Med. Jour., Jan. 24, 1903. [6] Ibid.

